# Supplementary material for: The testis protein ZNF165 is a SMAD3 cofactor that coordinates oncogenic TGFβ signaling in triple-negative breast cancer
Source: eLife. 2020 Jun 9;9:e57679. doi: 10.7554/eLife.57679 (PMC7302877; doi:10.7554/eLife.57679)
Supplement: Supplementary file 1. [file elife-57679-supp1.docx]

**Supplementary Table 1.** GREAT (ver. 3.0.0) analysis of the 118 ZNF165/SMAD3 co-bound regions determined using default association rules.

| Merged peak ID | Associated gene(s) and distance to merged peak (kb) |
| --- | --- |
| Merged-chr8-29513326-2 | DUSP4 (-305141), TMEM66 (+427397) |
| Merged-chr1-20940525-2 | PINK1 (-19423), CDA (+25084) |
| Merged-chr9-100459587-2 | XPA (+52) |
| Merged-chr9-86322733-2 | UBQLN1 (+385) |
| Merged-chr9-130007551-2 | ANGPTL2 (-122389), GARNL3 (-19254) |
| Merged-chr8-52921990-2 | PCMTD1 (-110332), ST18 (+400313) |
| Merged-chr5-36241864-3 | NADK2 (+59) |
| Merged-chr8-84664843-2 | RALYL (-432267) |
| Merged-chr11-14913531-2 | CYP2R1 (+267) |
| Merged-chr1-113498841-2 | SLC16A1 (-156) |
| Merged-chr19-3557712-2 | MFSD12 (-311) |
| Merged-chr13-76056696-2 | TBC1D4 (-446) |
| Merged-chr2-101179704-2 | PDCL3 (+552) |
| Merged-chr4-15683288-3 | FAM200B (-155) |
| Merged-chr11-576461-2 | PHRF1 (-60) |
| Merged-chr11-8932767-2 | ST5 (-269), AKIP1 (+80) |
| Merged-chr1-98511512-2 | SNX7 (-615767), DPYD (-124933) |
| Merged-chr6-74363968-2 | SLC17A5 (-90) |
| Merged-chr8-146052533-3 | ZNF7 (-440) |
| Merged-chr8-9912381-2 | MSRA (+603) |
| Merged-chr8-125487034-3 | RNF139 (-2) |
| Merged-chr8-101225325-2 | SPAG1 (+54669), RNF19A (+90162) |
| Merged-chr10-114221146-2 | TCF7L2 (-488863), VTI1A (+14130) |
| Merged-chr15-82337955-2 | MEX3B (+527) |
| Merged-chr2-96192422-2 | TRIM43 (-65344), FAHD2A (+123948) |
| Merged-chr15-79280960-2 | CTSH (-43527), RASGRF1 (+102155) |
| Merged-chr12-92539816-2 | BTG1 (-143) |
| Merged-chr12-62996780-3 | MON2 (+136237), PPM1H (+331983) |
| Merged-chr17-79651117-2 | ARL16 (-163), HGS (+155) |
| Merged-chr11-61880093-2 | FTH1 (-144961), INCENP (-11352) |
| Merged-chr19-2096427-2 | IZUMO4 (-454), MOB3A (-96) |
| Merged-chr19-18700069-2 | C19orf60 (+534) |
| Merged-chr20-49126770-2 | PTPN1 (-121) |
| Merged-chr20-47894862-3 | ZNFX1 (-178) |
| Merged-chrX-153687101-2 | PLXNA3 (+480) |
| Merged-chrX-153193640-2 | ARHGAP4 (-1926) |
| Merged-chr4-119757255-2 | SEC24D (+38) |
| Merged-chr6-143832560-2 | FUCA2 (+267) |
| Merged-chr8-8860330-2 | ERI1 (+673) |
| Merged-chr6-37400856-2 | FTSJD2 (-140) |
| Merged-chr21-35899190-2 | KCNE1 (-15577), RCAN1 (+88251) |
| Merged-chr21-46348409-2 | ITGB2 (+379) |
| Merged-chr6-152623424-2 | SYNE1 (+335110), ESR1 (+611793) |
| Merged-chr2-223906478-2 | KCNE4 (-10384), ACSL3 (+180826) |
| Merged-chr4-146540403-2 | MMAA (+988) |
| Merged-chr4-119274161-2 | PRSS12 (-3) |
| Merged-chr16-75278993-2 | BCAR1 (+20912), CTRB1 (+26095) |
| Merged-chr15-73076014-2 | ADPGK (+112) |
| Merged-chr2-66660885-2 | MEIS1 (-1647) |
| Merged-chr11-62554524-2 | TMEM179B (-375) |
| Merged-chr11-112097129-3 | PTS (+44) |
| Merged-chr15-51057675-2 | SPPL2A (+330) |
| Merged-chr16-88840447-2 | PIEZO1 (+11172), CTU2 (+67576) |
| Merged-chr19-50117852-2 | PRR12 (+22952), RRAS (+25606) |
| Merged-chr8-122101500-2 | SNTB1 (-275987), HAS2 (+552130) |
| Merged-chr2-133174192-2 | GPR39 (+45) |
| Merged-chr14-53982820-2 | DDHD1 (-363004), BMP4 (+440709) |
| Merged-chr10-94731901-2 | CYP26C1 (-89120), EXOC6 (+123624) |
| Merged-chr10-14920705-3 | SUV39H2 (-141) |
| Merged-chr10-88730332-3 | ADIRF (+2494), GLUD1 (+124180) |
| Merged-chr12-6982708-2 | SPSB2 (-208) |
| Merged-chr16-4526785-2 | NMRAL1 (-478), HMOX2 (+860) |
| Merged-chr16-2301613-2 | ECI1 (+2) |
| Merged-chr3-9811565-2 | CAMK1 (+96) |
| Merged-chr3-48343128-2 | NME6 (-280) |
| Merged-chr12-117256962-2 | HRK (+62284), RNFT2 (+80866) |
| Merged-chr11-2906935-2 | CDKN1C (+176) |
| Merged-chr14-24505721-2 | LRRC16B (-15488), DHRS4L2 (+47690) |
| Merged-chr14-92979854-2 | RIN3 (-264) |
| Merged-chr3-42642480-2 | NKTR (+374) |
| Merged-chr3-50374555-2 | TUSC2 (-8881), RASSF1 (+3717) |
| Merged-chr10-89621935-2 | PTEN (-935) |
| Merged-chr16-31483451-3 | TGFB1I1 (-4) |
| Merged-chr10-77161234-2 | ZNF503 (+430) |
| Merged-chr10-12238030-2 | CDC123 (+65), NUDT5 (+93) |
| Merged-chr5-7869628-2 | FASTKD3 (-513), MTRR (+411) |
| Merged-chr5-9546330-2 | SEMA5A (-143) |
| Merged-chr5-149829444-2 | RPS14 (-134) |
| Merged-chr12-12870172-3 | CDKN1B (+95) |
| Merged-chr7-158622497-2 | ESYT2 (-178) |
| Merged-chr14-23307642-2 | LRP10 (-33180), MMP14 (+1876) |
| Merged-chr1-54518939-2 | TCEANC2 (-335), TMEM59 (+172) |
| Merged-chr9-77703622-2 | NMRK1 (-497), OSTF1 (+163) |
| Merged-chr17-1994559-2 | SRR (-212439), HIC1 (+34955) |
| Merged-chr16-21313111-2 | CRYM (+1261), ANKS4B (+68125) |
| Merged-chr10-13390307-3 | SEPHS1 (+78) |
| Merged-chr10-104474036-3 | SFXN2 (-233), ARL3 (+100) |
| Merged-chr5-39074687-2 | RICTOR (-177) |
| Merged-chr4-101942078-2 | EMCN (-502828), PPP3CA (+326559) |
| Merged-chr10-88516050-2 | BMPR1A (-357) |
| Merged-chr5-171615431-2 | STK10 (-41) |
| Merged-chr10-75532353-2 | FUT11 (+304) |
| Merged-chrX-13416959-2 | EGFL6 (-170782), ATXN3L (-78441) |
| Merged-chr1-168148485-2 | TIPRL (+314) |
| Merged-chr2-54440092-2 | TSPYL6 (+43317), ACYP2 (+97451) |
| Merged-chr1-40723839-3 | ZMPSTE24 (-28) |
| Merged-chr7-73668808-2 | RFC2 (-34) |
| Merged-chr20-23618560-2 | CST3 (+22) |
| Merged-chr20-3788417-3 | AP5S1 (-12734), CDC25B (+11987) |
| Merged-chr1-110577313-2 | STRIP1 (+75) |
| Merged-chr20-33999887-2 | UQCC (+57) |
| Merged-chr22-32026534-2 | PISD (-2327) |
| Merged-chr16-74734689-2 | MLKL (+123) |
| Merged-chr12-125425089-2 | UBC (-25195), DHX37 (+48578) |
| Merged-chr20-35492149-3 | SOGA1 (-22) |
| Merged-chr20-23338630-2 | GZF1 (-4189) |
| Merged-chr6-160114377-3 | SOD2 (-105) |
| Merged-chr20-4048437-2 | SMOX (-80989), RNF24 (-52221) |
| Merged-chr2-26257054-2 | RAB10 (+75) |
| Merged-chr1-33282823-2 | S100PBP (-333), YARS (+931) |
| Merged-chr20-57267871-2 | NPEPL1 (+202) |
| Merged-chr17-73937052-2 | FBF1 (+67) |
| Merged-chr17-79008803-2 | BAIAP2 (-159) |
| Merged-chr1-201532066-2 | ENSG00000269690 (-59947), CSRP1 (-53482) |
| Merged-chr3-123553014-2 | PTPLB (-248982), MYLK (+50135) |
| Merged-chr1-201140010-2 | IGFN1 (-19943), TMEM9 (-16442) |
| Merged-chr1-44679250-2 | DMAP1 (+87) |
| Merged-chr8-138168647-2 | NONE |
