# Supplementary material for: The testis protein ZNF165 is a SMAD3 cofactor that coordinates oncogenic TGFβ signaling in triple-negative breast cancer
Source: eLife. 2020 Jun 9;9:e57679. doi: 10.7554/eLife.57679 (PMC7302877; doi:10.7554/eLife.57679)
Supplement: Supplementary file 2. [file elife-57679-supp2.docx]

**Supplementary Table 2.** Genes included in the EPITHELIAL_MESENCHYMAL_TRANSITION gene set with corresponding enrichment values for WHIM12 cells depleted of ZNF165.

| Gene | Rank in gene list | Rank metric score | Running ES |
| --- | --- | --- | --- |
| PMEPA1 | 13 | 0.56761646 | 0.03063989 |
| CXCL12 | 29 | 0.48706996 | 0.05673523 |
| VCAN | 44 | 0.44052756 | 0.08031484 |
| AREG | 86 | 0.35588926 | 0.09784545 |
| SLIT3 | 98 | 0.34782293 | 0.11646572 |
| MGP | 105 | 0.34447062 | 0.13515685 |
| WNT5A | 150 | 0.30529323 | 0.14974357 |
| VCAM1 | 226 | 0.26524693 | 0.160536 |
| COL1A1 | 250 | 0.25754473 | 0.1735635 |
| COL5A1 | 267 | 0.25123039 | 0.18660079 |
| ADAM12 | 286 | 0.24703117 | 0.1993042 |
| SCG2 | 295 | 0.24480994 | 0.2123966 |
| FGF2 | 308 | 0.24073581 | 0.22505972 |
| TNFRSF11B | 358 | 0.2274691 | 0.23509857 |
| COL7A1 | 374 | 0.22398758 | 0.24668454 |
| PTHLH | 425 | 0.21344571 | 0.25589883 |
| PDLIM4 | 489 | 0.20484717 | 0.26397392 |
| PLOD2 | 505 | 0.20218123 | 0.27435726 |
| FAS | 569 | 0.1927233 | 0.2817637 |
| DPYSL3 | 584 | 0.190909 | 0.2915765 |
| NID2 | 623 | 0.18694833 | 0.29994324 |
| MYLK | 714 | 0.17690566 | 0.30509624 |
| EDIL3 | 782 | 0.17062433 | 0.31107932 |
| CALD1 | 834 | 0.16699667 | 0.31768072 |
| SAT1 | 857 | 0.16488099 | 0.3256488 |
| SERPINE1 | 919 | 0.16013777 | 0.33136046 |
| IGFBP3 | 920 | 0.1601169 | 0.34019113 |
| THBS1 | 931 | 0.15950321 | 0.34847644 |
| CXCL1 | 1009 | 0.15408888 | 0.35303605 |
| FSTL3 | 1062 | 0.15091684 | 0.35869947 |
| PDGFRB | 1074 | 0.15024829 | 0.36642322 |
| TPM4 | 1177 | 0.1444156 | 0.36917055 |
| IL8 | 1232 | 0.14102399 | 0.37418607 |
| CTHRC1 | 1280 | 0.13797718 | 0.3793916 |
| THY1 | 1289 | 0.13760564 | 0.38657156 |
| GJA1 | 1296 | 0.13713425 | 0.3938278 |
| COL1A2 | 1420 | 0.1312076 | 0.39477253 |
| CDH11 | 1434 | 0.13018343 | 0.40128738 |
| MATN2 | 1495 | 0.12798633 | 0.40527695 |
| TAGLN | 1499 | 0.12780797 | 0.4121723 |
| IL6 | 1619 | 0.1224955 | 0.41284114 |
| ECM2 | 1720 | 0.11859886 | 0.41426694 |
| CALU | 1752 | 0.11723057 | 0.4191467 |
| FBLN2 | 1762 | 0.11675432 | 0.4251255 |
| FBLN5 | 1869 | 0.11298082 | 0.42593455 |
| GPC1 | 1874 | 0.11285032 | 0.43195382 |
| DCN | 1939 | 0.11025971 | 0.43476114 |
| CYR61 | 1945 | 0.11016192 | 0.44058096 |
| TPM2 | 2029 | 0.10741378 | 0.44225946 |
| FBN1 | 2248 | 0.10073538 | 0.43666425 |
| HTRA1 | 2539 | 0.09261261 | 0.42693824 |
| NTM | 2651 | 0.08958342 | 0.42620113 |
| COL16A1 | 2686 | 0.08879197 | 0.429359 |
| LAMC2 | 2910 | 0.0836659 | 0.42256665 |
| PRRX1 | 2968 | 0.0823506 | 0.4241928 |
| MEST | 3023 | 0.08075646 | 0.4258845 |
| SDC1 | 3175 | 0.07742483 | 0.42243078 |
| ITGB3 | 3206 | 0.07676163 | 0.42512977 |
| FBN2 | 3269 | 0.07553931 | 0.4261245 |
| SERPINH1 | 3454 | 0.07158833 | 0.42066094 |
| LRRC15 | 3480 | 0.07121859 | 0.42330998 |
| LUM | 3687 | 0.06681779 | 0.41645798 |
| BGN | 3935 | 0.06297076 | 0.40729666 |
| BMP1 | 3969 | 0.06246815 | 0.4090539 |
| COL5A2 | 4051 | 0.06099471 | 0.4082746 |
| GREM1 | 4263 | 0.05768948 | 0.4006634 |
| APLP1 | 4307 | 0.05708045 | 0.401612 |
| PCOLCE | 4384 | 0.05588669 | 0.40080675 |
| TFPI2 | 4395 | 0.05572226 | 0.4033684 |
| VEGFA | 4528 | 0.05374003 | 0.39958033 |
| PVR | 4593 | 0.05268149 | 0.39921212 |
| SLC6A8 | 4596 | 0.05267369 | 0.40201485 |
| SDC4 | 4701 | 0.05122623 | 0.39952037 |
| THBS2 | 4705 | 0.05113907 | 0.40218732 |
| IL15 | 4739 | 0.05055729 | 0.40328765 |
| TGM2 | 4814 | 0.04969549 | 0.40224326 |
| GEM | 4816 | 0.04967396 | 0.4049317 |
| TIMP3 | 4994 | 0.0473563 | 0.39848974 |
| SGCG | 5074 | 0.04636955 | 0.39700618 |
| RHOB | 5166 | 0.04503739 | 0.39483532 |
| SPOCK1 | 5247 | 0.04403411 | 0.3931718 |
| COL12A1 | 5254 | 0.04395448 | 0.39528903 |
| DKK1 | 5435 | 0.04171237 | 0.38838238 |
| MCM7 | 5479 | 0.04118946 | 0.38845453 |
| COL6A3 | 5505 | 0.04090776 | 0.3894319 |
| ITGAV | 5590 | 0.03979821 | 0.38733014 |
| CADM1 | 5706 | 0.0382573 | 0.38355774 |
| COPA | 5739 | 0.03775907 | 0.38400337 |
| VEGFC | 5757 | 0.03754098 | 0.38520426 |
| LOXL2 | 5815 | 0.03692221 | 0.38432497 |
| TGFB1 | 5919 | 0.03563181 | 0.38102156 |
| LEPRE1 | 5962 | 0.03503251 | 0.3808053 |
| POSTN | 5967 | 0.0349673 | 0.3825292 |
| FERMT2 | 5978 | 0.03483357 | 0.38393882 |
| ITGA2 | 6308 | 0.03067753 | 0.36880207 |
| BASP1 | 6525 | 0.02795035 | 0.35929498 |
| ABI3BP | 6720 | 0.02586109 | 0.35079798 |
| COL6A2 | 6759 | 0.0253917 | 0.35025465 |
| WIPF1 | 6783 | 0.02515183 | 0.35046533 |
| IGFBP4 | 6880 | 0.02404147 | 0.34688076 |
| TNC | 6893 | 0.02395297 | 0.347588 |
| GLIPR1 | 6908 | 0.02379484 | 0.3481842 |
| CDH2 | 7074 | 0.02197326 | 0.34095615 |
| MXRA5 | 7256 | 0.02024854 | 0.33281457 |
| SGCB | 7343 | 0.01931185 | 0.32948068 |
| TGFBI | 7503 | 0.01776267 | 0.32232735 |
| LAMC1 | 7611 | 0.0167065 | 0.31777558 |
| PPIB | 7629 | 0.01660875 | 0.317822 |
| FLNA | 7651 | 0.01641141 | 0.31765294 |
| VIM | 7742 | 0.01570217 | 0.31391537 |
| ITGB1 | 7776 | 0.01520756 | 0.3130661 |
| MYL9 | 7788 | 0.0150748 | 0.31333485 |
| FN1 | 7848 | 0.01442552 | 0.31111252 |
| SPARC | 7902 | 0.01384304 | 0.309165 |
| OXTR | 7987 | 0.01289087 | 0.30557927 |
| INHBA | 7999 | 0.01278106 | 0.3057215 |
| ANPEP | 8062 | 0.01218578 | 0.3032222 |
| SLIT2 | 8168 | 0.01105614 | 0.29846114 |
| SNAI2 | 8202 | 0.0107495 | 0.297366 |
| COL5A3 | 8239 | 0.01028519 | 0.2960918 |
| CD44 | 8281 | 0.00987372 | 0.29453918 |
| EFEMP2 | 8422 | 0.00852813 | 0.28784838 |
| COL3A1 | 8441 | 0.00831281 | 0.28738612 |
| MFAP5 | 8578 | 0.00698446 | 0.2808148 |
| ECM1 | 8581 | 0.00696354 | 0.28109655 |
| FOXC2 | 8808 | 0.00481798 | 0.26980218 |
| ITGB5 | 8922 | 0.00384721 | 0.2642343 |
| FBLN1 | 8978 | 0.00327367 | 0.26160154 |
| GLT25D1 | 9017 | 0.00289967 | 0.25981775 |
| COL11A1 | 9174 | 0.00138571 | 0.25191462 |
| PLOD1 | 9252 | 6.66E-04 | 0.2480127 |
| CTGF | 9328 | -8.50E-05 | 0.24418108 |
| CAP2 | 9474 | -0.0013422 | 0.23683822 |
| LGALS1 | 9486 | -0.0014449 | 0.23635526 |
| PLOD3 | 9519 | -0.0016581 | 0.23480988 |
| BDNF | 9563 | -0.0020546 | 0.2327237 |
| FSTL1 | 9606 | -0.0024699 | 0.23071158 |
| SGCD | 9653 | -0.0029307 | 0.22852027 |
| LAMA1 | 9709 | -0.0034323 | 0.22589627 |
| TIMP1 | 9851 | -0.0048469 | 0.2189513 |
| ELN | 10333 | -0.0093318 | 0.19486238 |
| FZD8 | 10737 | -0.0131242 | 0.1749724 |
| NNMT | 10752 | -0.0132451 | 0.17498676 |
| ITGA5 | 10969 | -0.0151736 | 0.16477503 |
| LRP1 | 11193 | -0.0172873 | 0.15432179 |
| LOX | 11407 | -0.0192149 | 0.14448638 |
| MMP2 | 11452 | -0.0195715 | 0.14331514 |
| EMP3 | 11536 | -0.0202871 | 0.14018847 |
| CXCL6 | 11624 | -0.0209814 | 0.13689551 |
| CD59 | 12034 | -0.0249881 | 0.11735292 |
| NT5E | 12456 | -0.0292133 | 0.09742954 |
| ENO2 | 12597 | -0.0307043 | 0.09196181 |
| DAB2 | 12724 | -0.0319942 | 0.08728132 |
| JUN | 12918 | -0.0339807 | 0.07928328 |
| IGFBP2 | 13186 | -0.0369693 | 0.0676649 |
| SERPINE2 | 13425 | -0.0398277 | 0.05768754 |
| SFRP4 | 13729 | -0.0430994 | 0.04456582 |
| FAP | 14015 | -0.0466605 | 0.0325612 |
| GPX7 | 14095 | -0.0477311 | 0.03115272 |
| NOTCH2 | 14130 | -0.048085 | 0.03206554 |
| COL4A1 | 14380 | -0.0512702 | 0.02215659 |
| CRLF1 | 14402 | -0.051462 | 0.02392062 |
| PFN2 | 14418 | -0.0516793 | 0.02600354 |
| CAPG | 14471 | -0.0523547 | 0.02623113 |
| COL4A2 | 14645 | -0.0547639 | 0.02040233 |
| QSOX1 | 14758 | -0.0564156 | 0.01778483 |
| COMP | 14788 | -0.0567131 | 0.01942927 |
| GADD45A | 14796 | -0.0568877 | 0.02220865 |
| FUCA1 | 15144 | -0.0614782 | 0.0078499 |
| PLAUR | 15210 | -0.0622023 | 0.00795564 |
| SPP1 | 15231 | -0.0624753 | 0.01037822 |
| RGS4 | 15258 | -0.0627429 | 0.01250866 |
| ID2 | 15440 | -0.0654835 | 0.00686185 |
| COL8A2 | 15513 | -0.0664578 | 0.00684423 |
| ACTA2 | 15959 | -0.0741154 | -0.0118304 |
| GADD45B | 16023 | -0.0753321 | -0.0108982 |
| GAS1 | 16048 | -0.0758099 | -0.0079448 |
| PMP22 | 16269 | -0.0796716 | -0.014804 |
| IL32 | 16673 | -0.0879856 | -0.0305653 |
| MSX1 | 16737 | -0.0893166 | -0.0288618 |
| TNFRSF12A | 16829 | -0.0908614 | -0.0285054 |
| MMP14 | 16969 | -0.0939214 | -0.0304355 |
| LOXL1 | 17140 | -0.0977659 | -0.0337392 |
| TGFBR3 | 17183 | -0.0986952 | -0.0304444 |
| SNTB1 | 17235 | -0.0999406 | -0.0275412 |
| MAGEE1 | 18226 | -0.1330296 | -0.0708438 |
| CDH6 | 18267 | -0.1350486 | -0.0654417 |
| PCOLCE2 | 19070 | -0.1878713 | -0.0961034 |
| TNFAIP3 | 19115 | -0.192726 | -0.0877249 |
| PTX3 | 19333 | -0.2237246 | -0.0864859 |
| MATN3 | 19386 | -0.2318367 | -0.0763597 |
| SFRP1 | 19566 | -0.2855656 | -0.0697663 |
| LAMA3 | 19652 | -0.3347397 | -0.0556528 |
| MMP1 | 19719 | -0.462491 | -0.0335217 |
| MMP3 | 19737 | -0.6300683 | 3.58E-04 |
